# The Combination of Graphene and Polycaprolactone Scaffolds Enhancing Bone Mineralization and Hydroxyapatite

**DOI:** 10.1055/s-0045-1809145

**Published:** 2025-05-27

**Authors:** Silvia Anitasari, Nataniel Tandirogang, Hendrik Setia Budi, Yung-Kang Shen, Hadi Irawiraman, Marut Tangwattanachuleeporn

**Affiliations:** 1 Department of Dental Materials and Devices, Dentistry Program, Faculty of Medicine, Universitas Mulawarman, Samarinda, Indonesia; 2 Department of Medical Microbiology, Medical Program, Faculty of Medicine, Universitas Mulawarman, Samarinda, Indonesia; 3 Department of Oral Biology, Faculty of Dental Medicine, Universitas Airlangga, Surabaya, Indonesia; 4 School of Dental Technology, College of Oral Medicine, Taipei Medical University, Taipei, Taiwan; 5 Department of Anatomical Pathology, Faculty of Medicine, Universitas Mulawarman, Samarinda, Indonesia; 6 Department of Medical Sciences, Faculty of Allied Health Science, Burapha University, Chonburi, Thailand; 7 Research Unit for Sensor Innovation, Burapha University, Chonburi, Thailand

**Keywords:** polycaprolactone, graphene, mineralization, bone regeneration

## Abstract

**Objective:**

This study aimed to evaluate the effects of incorporating varying concentrations of graphene (0.5, 1.5, and 2.5 wt%) into polycaprolactone (PCL) scaffolds on mineralization and hydroxyapatite formation for bone tissue engineering applications.

**Materials and Methods:**

PCL scaffolds were fabricated with three different graphene concentrations: 0.5, 1.5, and 2.5 wt%. The scaffolds underwent characterization using Fourier-transform infrared spectroscopy (FTIR) to assess chemical composition and mineralization. Radiological imaging was employed to evaluate structural integrity and mineral density over a 21-day period. Additionally, histology analysis was performed to assess cellular interactions and scaffold integration.

**Results:**

FTIR analysis on day 7 indicated early mineralization across all scaffolds, evidenced by phosphate (∼1030 cm ^−1^ ) and hydroxyl (∼3500 cm ^−1^ ) peaks, suggesting initial hydroxyapatite deposition. By day 21, the 2.5 wt% graphene scaffold demonstrated the highest degree of mineralization, with significantly increased hydroxyapatite formation compared with the other groups. However, this scaffold also exhibited signs of degradation, implying that higher graphene concentrations might compromise long-term scaffold stability. The 1.5 wt% graphene scaffold showed consistent mineralization and favorable osteoconductivity but did not reach the mineral deposition levels observed in the 2.5 wt% group.

**Conclusion:**

Incorporating graphene into PCL scaffolds enhances mineralization and hydroxyapatite formation, with the 2.5 wt% concentration achieving the most substantial effects. The 2.5 wt% graphene scaffold presents a balanced alternative, promoting steady mineralization and maintaining structural integrity, making it a promising candidate for bone tissue engineering applications.

## Introduction

The bone matrix plays a pivotal role in influencing the mineralization process by osteoblasts through various signaling pathways and bioactive components, including hydroxyapatite. Osteoblasts are the primary cells responsible for bone formation. These cells actively interact with the extracellular matrix (ECM), which not only provides structural support but also delivers essential biochemical signals for osteoblast differentiation and function, thus supporting bone tissue regeneration. 1 2

In recent years, graphene has attracted increasing attention in the field of bone tissue engineering due to its unique properties. 3 Graphene is known for its exceptional mechanical strength, high electrical conductivity, and large surface area. These characteristics make graphene a promising material for enhancing the performance of scaffolds used in bone regeneration. 2 4 The integration of graphene into polymer-based scaffold materials, such as polycaprolactone (PCL), has been shown to improve both the mechanical properties and bioactivity of the scaffold, thereby creating an optimal microenvironment for osteoblast differentiation and mineralization.

One of the key mechanisms by which graphene enhances mineralization is its ability to facilitate the adsorption of calcium and phosphate ion, which are essential for hydroxyapatite formation. 4 The high surface area of graphene promotes the nucleation and growth of mineral deposits on the scaffold surface, essential for osteogenesis and bone tissue development. Additionally, several studies have shown that graphene can enhance the osteogenic differentiation of stem cells, such as mesenchymal stem cells, by improving cell adhesion, proliferation, and the expression of osteogenic markers. 5 6

Furthermore, the incorporation of graphene into polymeric scaffolds, such as PCL, has been shown to improve both the mechanical properties and mineralization potential of the scaffolds. Graphene provides a framework for hydroxyapatite crystal growth, improving structural integrity while promoting biological processes crucial for effective bone regeneration. 7 8 Moreover, graphene exhibits antimicrobial properties that provide as additional layer of protection against bacterial infections during the healing process, making it a promising candidate for bone tissue engineering applications 1 8

The study aims to explore the effect of graphene on the mineralization process in bone tissue engineering, with a particular focus on the role of hydroxyapatite as a key component in bone formation. It also seeks to understand how the integration of graphene into PCL scaffolds can promote ECM mineralization and support effective bone regeneration. Additionally, the study will investigate the impact of various graphene concentration (0.5, 1.5, and 2.5 wt%) on the mineralization process and their effect on osteogenesis.

## Materials and Methods

### Fabrication PCL/Graphene Scaffold

A solvent casting and particle leaching method was used to fabricate PCL and PCL/graphene (G) scaffolds. PCL (Sigma-Aldrich, Darmstadt, Germany) was dissolved in chloroform (Honeywell, Charlotte, United States) at room temperature for 12 hours. This combination was then mixed with various concentrations of G and NaCl (Sigma-Aldrich, Darmstadt, Germany) for 2 hours. G was previously produced by transferring a graphite intercalation compound into a preheated crucible at 700°C in a common furnace positioned in the front of a fume cupboard to prevent inhalation of the nanoparticles, and it was left there for 60 seconds. These layers expanded upon ultrasonication and caused the G to disperse in the solvent. After fabrication, the blend was placed into a cast and cured overnight at room temperature. Chloroform was then evaporated for 24 hours at 37°C in a drying vacuum oven (Deng Yng, Taipei, Taiwan). Deionized (DI) water and a water bath (BH-130D, Taipei, Taiwan) were used to remove porogen from the scaffold. In addition, the DI water was changed every 2 hours and then dried in the oven at 50°C for 12 hours. 9 10

### Preparation of Bone Defect Animal Model

The research was conducted with ethical clearance from Faculty of Medicine, Mulawarman University (no. 191/KEPK-FK/VII/2024) using rabbit animal models, as defects can be made sufficiently large. The rabbits were placed in individual cages under standard laboratory conditions (temperature 25 ± 10°C, humidity 55 ± 7%). Each rabbit was fed *ad libitum* (water and pellets). A scaffold measuring 4 × 4 × 4 mm ^3^ with various graphene weight ratios was sterilized in 95% ethanol for 24 hours. The scaffold was then rinsed three times with phosphate-buffered saline 1× and ultraviolet-sterilized for 60 minutes before implantation.

The surgery was performed under general anesthesia, consisting of a mixture of 0.35 mL/kg ketamine chlorhydrate (Benofarm, Jakarta, Indonesia) and 0.25 mL/kg xylazine chlorhydrate (Bimeda, Dublin, Ireland). The first step of the surgery is to make an incision and create a flap on the left side of the tibia, where the defect will be located. A standardized transverse tibial osteotomy was performed with a width of 5 mm, length of 5 mm, and depth of 1.5 mm using a surgical burr (Hogy, Jakarta, Indonesia) to create the bone defect. Afterward, the sterilized scaffold was placed inside the defect and the flap was closed and secured using Vicryl 6.0 sutures (Ethicon, California, United States).

The rabbits were monitored postsurgery and kept under standard conditions throughout the healing period. On day 7, before euthanasia, radiographic imaging was performed on the left tibia bone of the rabbit to capture the state of the bone defect and scaffold implantation. The euthanasia and necropsy procedure followed. The tibia bone was sectioned at 5 μm a microtome for further analysis. Fourier transform infrared (FTIR) and hematoxylin and eosin (H&E) analyses were conducted to assess osteogenesis on days 7, 14, and 21, while radiographic imaging was performed only on days 7 and 21. 11

### Radiographic Analysis

Carefully position the rabbit's leg on the cone of the radiograph machine (YSDR-Vet320, Siemens, Germany) ensuring proper alignment to capture the targeted area. Secure the leg gently to minimize movement and avoid stress to the animal. 12

Set the radiograph machine's parameters, including the appropriate exposure time. For small animals like rabbits, an exposure time of approximately 0.05 to 0.1 seconds is typically sufficient, depending on the machine and the desired image quality. Take the radiograph by activating the machine to capture the image. Use the machine's built-in image enhancement features to improve the quality. 13

The interpretation of the radiographic image focused on assessing bone integration and scaffold performance at days 7 and 21. Bone formation was analyzed by observing changes in radiopacity. The radiographic images were then compared across different experimental groups to evaluate the effect of varying graphene concentrations (0.5, 1.5, and 2.5 wt% G) of bone regeneration. 6 12

### FTIR Analysis

Bone samples undergoing demineralization and mineralization processes were placed in front of an FTIR crystal (Shimadzu, Japan). The infrared light, which serves as the light source, is divided into two beams: one passes through the sample and the other through a reference. Both beams sequentially pass through the FTIR crystal, and the signal is captured by a detector, then converted into an electrical signal and recorded by a recorder. The wavelength range used for measurement is 500 to 4000 cm ^−1^ (wavenumber). 14 To interpret FTIR results, absorption peaks and their intensity at specific wavenumbers should be identified. Then, the spectra from different time points (7, 14, and 21days) can be compared to evaluate changes in mineralization over time. 8 11

### Histology Analysis

The fixed tissue is washed with distilled water and soaked in Biodec R (Thermo Fisher, Pittsburgh, United States) for 5 days at room temperature (decalcification process). H&E staining (Thermo Fisher) was performed on the tissue to observe osteoblast, osteoclast, and osteocytes. 15

## Results

### Radiograph Analysis

**Fig. 1:**
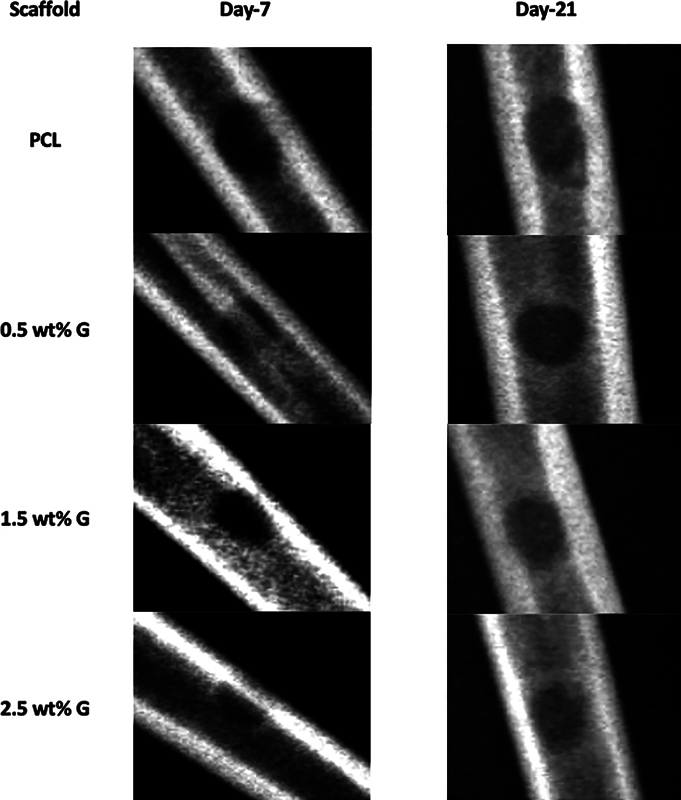
Radiograph of the bone of the rabbit on the days 7 and 21 after implantation of polycaprolactone/graphene (PCL/G) scaffolds.

On day 7, for the PCL, the radiograph showed minimal bone integration with the scaffold. The edges of the scaffold appear intact, but limited signs of new bone formation or osseointegration were visible. Slight inflammation or soft tissue swelling might have been present. For the 0.5 wt% G, early signs of bone formation around the scaffold were noticeable. Radiopacity near the implant edges suggested mild osteogenic activity, but it was not significantly different from the PCL. For 1.5 wt% G, enhanced radiopacity near the scaffold suggested more pronounced bone regeneration compared with the PCL and 0.5 wt% G scaffold. Initial trabecular bone growth was visible at the interface between the scaffold and the host rabbit's tissue. For 2.5 wt% G, the radiograph indicated more substantial early bone formation. However, slight irregularities in the implant's edges may have suggested potential stress or degradation at this concentration, as shown in Fig. 1 . 16

On day 21, for the PCL, the radiograph showed moderate bone integration with the scaffold. Some trabecular bone formation was evident, but overall osseointegration remained inferior compared with scaffolds with graphene. For 0.5 wt% G, the radiograph showed improved radiopacity compared with day 7, with moderate bone formation extending into the scaffold structure. Bone growth remained consistent but less significant than higher graphene concentrations. For 1.5 wt% G, significant new bone formation and clear osseointegration were observed. Radiopacity highlighted dense, well-formed trabecular bone surrounding and infiltrating the scaffold. This concentration showed optimal performance in terms of biocompatibility and osteogenesis. For the 2.5 wt% G, while radiopacity indicated extensive bone formation, minor signs of scaffold degradation or stress fracture were visible. This may have suggested that a higher graphene concentration could compromise mechanical over time, as shown in Fig. 1 . 17 18

### Fourier Transform Infrared Spectroscopy Analysis

**Fig. 2:**
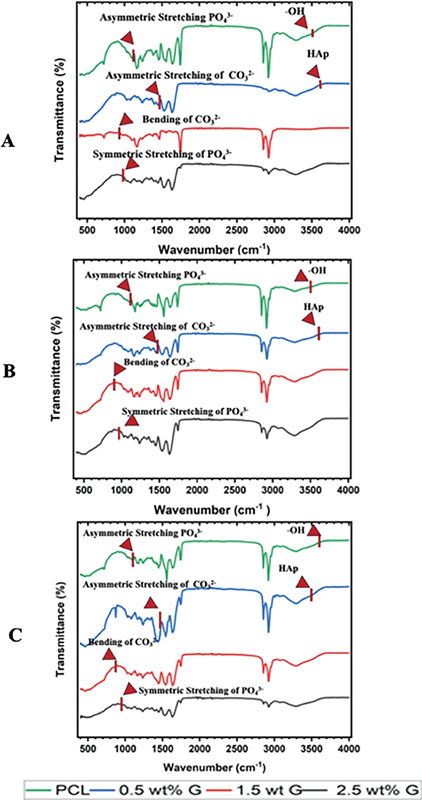
Fourier transform infrared spectroscopy of the rabbit bone on days 7 ( **A** ), 14 ( **B** ), and 21 ( **C** ) after implantation of polycaprolactone/graphene (PCL/G) scaffolds.

On the day 7, for PCL, the FTIR spectra showed characteristic PCL peaks (e.g., carbonyl stretching at ∼1720 cm ^−1^ ) with no significant changes related to mineral deposition. This suggests minimal interaction with the biological environment and negligible hydroxyapatite formation within the first week. However, on day 21, slight reductions in PCL peak intensities could indicate partial scaffold degradation. No strong evidence of hydroxyapatite formation was detected, as there were no new peaks associated with the phosphate groups (∼1000–1100 cm ^−1^ ), carbonate group (∼1410–1460 cm ^−1^ ), and hydroxyl groups (∼3500 cm ^−1^ ), as shown in Fig. 2A and C .

On day 7, for 0.5 wt% G, the FTIR spectra showed weak signs of phosphate-related peaks (∼1030 cm ^−1^ ) and hydroxyl stretching (∼3500 cm ^−1^ ), suggesting initial stages of hydroxyapatite deposition. This indicated that the addition of graphene enhanced scaffold interactions with the surrounding biological fluids. Moreover, the phosphate and hydroxyl peaks became more pronounced on day 21, indicating increased mineralization and hydroxyapatite formation. However, the mineralization extent was moderate due to the low graphene content, which limits scaffold bioactivity, as shown in Fig. 2A and C . 19 20

On day 7, the 1.5 wt% G scaffold exhibited noticeable phosphate (∼1030 cm ^−1^ ) and hydroxyl (∼3500 cm ^−1^ ) peaks in the FTIR spectra, signaling the onset of hydroxyapatite formation. This balanced graphene concentration facilitated early mineralization while maintaining scaffold stability, as shown in Fig. 2A . By day 14, the scaffold demonstrated a continued and consistent increase in hydroxyapatite (3100–3600 cm ^−1^ ) deposition, with stronger phosphate and hydroxyl peaks, indicating progressive mineralization. The graphene content at this level provided optimal nucleation sites for hydroxyapatite crystallization, enhancing the bioactivity and osteoconductivity of the scaffold, as shown in Fig. 2B .

On day 21, the 1.5 wt% G scaffold showed significant hydroxyapatite deposition, as evidenced by the heightened intensity of phosphate and hydroxyl peaks in the spectra. The consistent mineralization performance demonstrated its ability to support robust osteogenesis without compromising scaffold stability, as shown in Fig. 2C . However, until day 21, the scaffold did not exhibit noticeable signs of degradation, which could be a limitation in balancing mineralization with the gradual replacement of the scaffold by natural bone tissue. Although the 1.5 wt% G scaffold provides a steady mineralization rate and favorable bioactivity.

On day 7, for 2.5 wt% G, the spectra showed clear and prominent evidence of phosphate (∼1030 cm ^−1^ ) and hydroxyl (∼3500 cm ^−1^ ) groups, indicating rapid and substantial initial mineralization. The high graphene content created an enhanced bioactive surface, accelerating the nucleation and growth of hydroxyapatite crystals (∼3200–3600 cm ^−1^ ), as shown in Fig. 2A . While some interference in the PCL matrix was noted due to the elevated graphene concentration, this did not significantly hinder the scaffold's ability to support mineralization. By day 14, the FTIR spectra revealed a marked increase in hydroxyapatite deposition, with pronounced phosphate and hydroxyl peaks reflecting the scaffold's strong osteoconductive properties, as shown in Fig. 2B . The higher graphene content appeared to amplify mineralization, creating an ideal microenvironment for osteoblast activity and mineral deposition. On day 21, the 2.5 wt% G scaffold exhibited the most robust mineralization, with enhanced intensity of phosphate and hydroxyl peaks, confirming significant hydroxyapatite formation.

### Histology Analysis

**Fig. 3:**
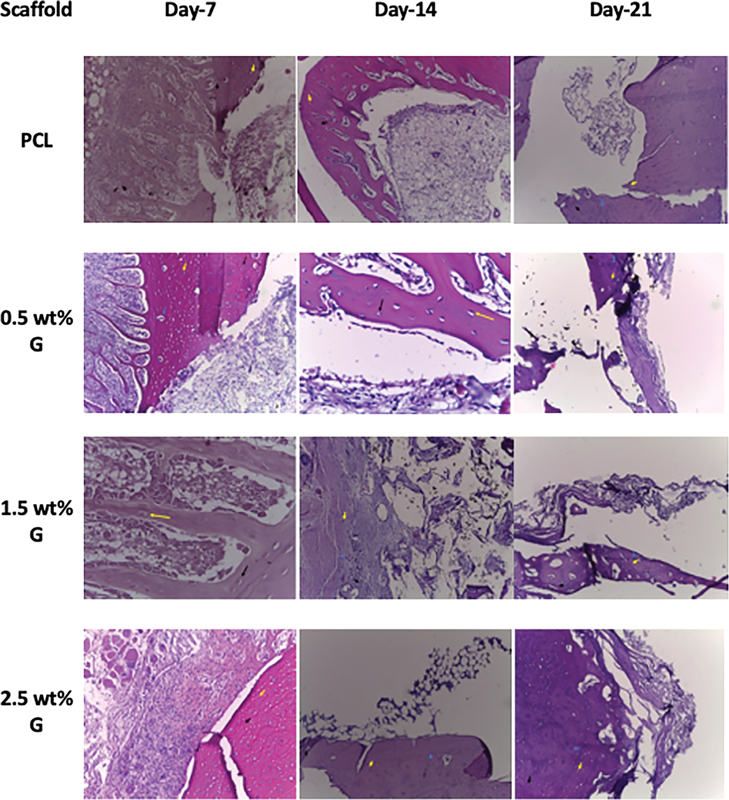
Hematoxylin and eosin staining of rabbit bone after implantation of polycaprolactone/graphene (PCL/G) scaffold on days 7, 14, and 21. The image shows osteocytes (  ), osteoblast (  ), and osteoclast (  ) in the area surrounding the bone defect.

At day 7, a mild tissue reaction was observed, characterized by a moderate presence of inflammatory cells including macrophage and neutrophils. Vascularization around the scaffold was limited, and the scaffold remained largely unintegrated with the surrounding tissue. In the 1.5 wt% G, cellular infiltration was slightly improved compared with the 0.5 wt% G group, with the development of new blood vessels and early signs of fibroblast proliferation. The 2.5 wt% G showed a more pronounced inflammatory response, accompanied by increased vascularization and heightened fibroblast activity. Some integration of the scaffold with the surrounding tissue was evident, suggesting an early biological response to the material, as shown in Fig. 3 . 19 21

At day 14, for PCL scaffolds, tissue integration was minimal and inflammatory cells persisted around the implant. The 0.5 wt% G exhibited moderate collagen deposition and partial integration with the surrounding tissue. The 1.5 wt% G demonstrated significant tissue integration, visible collagen formation, and the presence of osteoblast. In 2.5 wt% G, collagen deposition was more extensive, inflammation was notably reduced, and osteogenesis was significantly enhanced, as shown in Fig. 3 .

At day 21, PCL exhibited minimal bone regeneration, with inflammatory markers still present. The 0.5 wt% G showed improved bone tissue formation, although fibrous tissue encapsulation was observed in some areas. The 1.5 wt% G displayed substantial bone tissue formation with well-organized structure. The 2.5 wt% G demonstrated superior bone regeneration, characterized by the formation of organized lamellar bone and minimal inflammation, as shown in Fig. 3 .

## Discussion

At a 2.5 wt% G concentration, the inclusion of graphene significantly enhanced the scaffold's ability to provide optimal nucleation sites for the formation of hydroxyapatite, a key component in bone mineralization. This optimal level of graphene content facilitated a favorable microenvironment for mineral deposition, resulting in enhanced mineralization, as evidenced by the pronounced phosphate and hydroxyl peaks observed in the FTIR spectra. These peaks are indicative of the formation of bone-like apatite, which is essential for the integration of the scaffold into bone tissue and supporting the process of bone regeneration. 5 Specifically, the presence of phosphate and hydroxyl groups in the FTIR spectra strongly suggests that a mineralized structure similar to natural bone was formed, further validating the role of graphene in enhancing scaffold functionality and promoting osteogenesis. 2 22

The FTIR analysis also provided valuable insights into the dynamic relationship between scaffold degradation and hydroxyapatite formation. PCL, without graphene, degraded without significant mineralization, which limits its ability to facilitate bone regeneration. In contrast, the addition of graphene, particularly at a concentration of 2.5 wt%, stabilized the PCL scaffold while promoting the deposition of hydroxyapatite. This not only enhanced the mineralization process but also helped in retaining the structural integrity of the scaffold, a critical factor for successful bone tissue engineering applications. Graphene's ability to support hydroxyapatite formation and scaffold stability underscores its pivotal role in improving the overall performance of the scaffold during degradation and mineralization. 13 16

When graphene concentrations were increased, especially at 1.5 and 2.5 wt%, a more significant promotion of osteogenesis was observed, driven by the superior properties conferred to the scaffolds, such as enhanced mechanical strength, increased electrical conductivity, and improved bioactivity. 23 These properties are crucial for promoting osteoblasts activity, cellular attachment, and mineral deposition, all of which are integral to the osteogenic process. 19 The increased mechanical strength ensures that the scaffold can withstand the forces encountered in the body while providing a stable matrix for cell growth and tissue formation. 24 The electrical conductivity of graphene, in particular, has been shown to promote cell signaling and accelerate the differentiation of osteoblasts, thereby enhancing bone formation. 15 Furthermore, graphene's inherent bioactivity facilitates the interaction with cells, promoting a more favorable environment for bone regeneration. 11 25

In contrast, PCL scaffolds without graphene exhibited comparatively lower osteogenic potential, demonstrating that the incorporation of graphene plays a critical role in improving the biological performance of the scaffold. 26 This finding is consistent with other studies that have shown that graphene and its derivatives can significantly enhance bone regeneration by improving scaffold properties and promoting osteogenic activity. The reduced osteogenic performance of PCL scaffolds highlights the need for advanced composite materials, such as PCL/G, to achieve optimal outcomes in bone tissue engineering. 4 8

The synergetic effects of graphene concentration on osteogenesis were further confirmed through various analyses, including H&E staining, radiographic imaging, and FTIR analysis. Radiographic imaging provided visual confirmation of the mineralization and structural integrity of the scaffolds, showing that scaffolds with higher graphene concentration exhibited enhanced bone-like mineralization. These findings, in combination with the FTIR analysis, provide strong evidence for the enhanced osteogenic potential of PCL/G composite scaffolds. 17 27

These collective findings emphasize the critical role of graphene in improving both the chemical and biological properties of scaffolds, and they highlight the potential of PCL/G composites for bone regeneration applications. 28 29 The optimized concentration of 2.5 wt% G represents a balance between providing sufficient nucleation sites for hydroxyapatite formation and maintaining the structural integrity of the scaffold. By enhancing the mineralization process and promoting osteoblast activity, these composite scaffolds offer a promoting solution for bone defect repair and regeneration. 2 30 This research not only underscores the importance of graphene in biomaterial design but also provides insights into the optimizing of composites scaffolds for enhanced bone repair, paving the way for future advances in tissue engineering and regenerative medicine. 2 31

## Conclusion

The scaffolds' ability to promote mineralization, as validated by all three techniques, directly supports osteogenesis. This is most pronounced in scaffolds with higher graphene concentrations, highlighting graphene's role in enhancing mineralization, which is essential for successful bone regeneration.
